# Supplementary material for: Association of Postpartum Depression with Maternal Suicide: A Nationwide Population-Based Study
Source: Int J Environ Res Public Health. 2022 Apr 23;19(9):5118. doi: 10.3390/ijerph19095118 (PMC9099720; doi:10.3390/ijerph19095118)
Supplement: Supplementary file 1 [file ijerph-19-05118-s001.zip › ijerph-1627549-supplementary.pdf]

Supplementary Table S1. Characteristics of study in the baseline

Cohort	Delivery, with PPD		Delivery, without PPD			Without delivery		
**Variables**	*n*	%		%	*p*	*n*	%	*p*
**Total**	2,882	33.33	5,764	66.67		5,764	66.67	
**Age (years)**	28.31 ± 4.84		28.20 ± 5.32		0.351	28.25 ± 5.35		0.612
**Age groups (yrs)**					0.999			0.999
≦20	188	6.52	376	6.52		376	6.52	
21-30	1,785	61.94	3,570	61.94		3,570	61.94	
31-34	619	21.48	1,238	21.48		1,238	21.48	
35-37	185	6.42	370	6.42		370	6.42	
38-40	81	2.81	162	2.81		162	2.81	
41-43	18	0.62	36	0.62		36	0.62	
≧44	6	0.21	12	0.21		12	0.21	
**Insured premium (NT$)**					0.991			0.979
<18,000	2,527	87.68	5,052	87.65		5,060	87.79	
18,000-34,999	247	8.57	498	8.64		493	8.55	
≧35,000	108	3.75	214	3.71		211	3.66	
**HTN**					0.002			0.001
Without	2,877	99.83	5,726	99.34		5,722	99.27	
With	5	0.17	38	0.66		42	0.73	
**DM**					<0.001			0.024
Without	2,869	99.55	5,683	98.59		5,712	99.10	
With	13	0.45	81	1.41		52	0.90	
**Hyperlipidemia**					0.002			0.007
Without	2,881	99.97	5,742	99.62		5,745	99.67	
With	1	0.03	22	0.38		19	0.33	
**COPD**					<0.001			<0.001
Without	2,879	99.90	5,716	99.17		5,709	99.05	
With	3	0.10	48	0.83		55	0.95	
**CKD**					0.844			0.493
Without	2,878	99.86	5,755	99.84		5,759	99.91	
With	4	0.14	9	0.16		5	0.09	
**IHD**					<0.001			<0.001
Without	2,875	99.76	5,655	98.11		5,643	97.90	
With	7	0.24	109	1.89		121	2.10	
**CHD**					0.671			0.002
Without	2,881	99.97	5,759	99.91		5,742	99.62	
With	1	0.03	5	0.09		22	0.38	
**Stroke**					0.004			0.009

Without	2,879	99.90	5,736	99.51		5,738	99.55	
With	3	0.10	28	0.49		26	0.45	
**Cancer**					<0.001			<0.001
Without	2,877	99.83	5,666	98.30		5,626	97.61	
With	5	0.17	98	1.70		138	2.39	
**Obesity**					0.617			0.671
Without	2,881	99.97	5,763	99.98		5,760	99.93	
With	1	0.03	1	0.02		4	0.07	
**Season**					0.999			0.999
Spring (Mar-May)	598	20.75	1,196	20.75		1,196	20.75	
Summer (Jun-Aug)	811	28.14	1,622	28.14		1,622	28.14	
Autumn (Sep-Nov)	864	29.98	1,728	29.98		1,728	29.98	
Winter (Dec-Feb)	609	21.13	1,218	21.13		1,218	21.13	
**Location**					<0.001			<0.001
Northern Taiwan	1,290	44.76	2,625	45.54		2,635	45.71	
Middle Taiwan	775	26.89	1,677	29.09		1,829	31.73	
Southern Taiwan	688	23.87	1,142	19.81		976	16.93	
Eastern Taiwan	108	3.75	304	5.27		291	5.05	
Outlets islands	21	0.73	16	0.28		33	0.57	
**Urbanization level**					<0.001			<0.001
1 (The highest)	878	30.46	2,155	37.39		2,064	35.81	
2	1,215	42.16	2,497	43.32		2,533	43.95	
3	398	13.81	538	9.33		577	10.01	
4 (The lowest)	391	13.57	574	9.96		590	10.24	
**Level of care**					<0.001			<0.001
Hospital center	521	18.08	2,018	35.01		2,045	35.48	
Regional hospital	733	25.43	2,271	39.40		2,457	42.63	
Local hospital	1,628	56.49	1,475	25.59		1,262	21.89	

***P*: Chi-square/Fisher exact test on category variables and t-test on continue variables**

Supplementary Table S2. Years to suicide

Cohort	Min	Median	Max	Mean ± SD
Delivery, with PPD *(Study cohort)*	0.05	0.98	12.72	1.61 ± 2.18
Delivery, without PPD *(Comparison cohort 1)*	0.79	5.12	10.31	4.87 ± 2.88
Without delivery (*Comparison cohort 2)*	0.17	4.26	8.32	4.16 ± 2.35
Overall	0.05	1.14	12.72	2.14 ± 2.52

Supplementary Table S3. Factors of suicide by using Cox regression

Variables	Crude HR	95% CI	95% CI	*p*	Adjusted HR	95% CI	95% CI	*p*
**Cohort**								
Delivery, with PPD *(Study cohort)*	28.065	8.848	89.016	<0.001	20.051	6.210	64.733	<0.001
Delivery, without PPD *(Comparison cohort 1)*	1.198	0.268	5.354	0.789	1.093	0.244	4.907	0.882
Without delivery (*Comparison cohort 2)*	Reference				Reference			
**Age groups (yrs)**								
≦20	Reference				Reference			
21-30	0.404	0.160	1.017	0.057	0.598	0.234	1.527	0.291
31-34	0.251	0.093	0.676	0.007	0.427	0.154	1.183	0.106
35-37	0.212	0.071	0.638	0.006	0.497	0.161	1.542	0.232
38-40	0.135	0.039	0.471	0.002	0.200	0.056	0.711	0.014
41-43	0.179	0.047	0.668	0.011	0.219	0.057	0.849	0.029
≧44	0.182	0.066	0.496	0.001	0.178	0.063	0.501	0.001
**Insured premium (NT$)**								
<18,000	Reference				Reference			
18,000-34,999	0.627	0.088	4.497	0.644	0.558	0.072	4.323	0.577
≧35,000	1.948	0.271	13.967	0.493	4.235	0.548	32.737	0.161
**HTN**								
Without	Reference				Reference			
With	0.172	0.024	1.228	0.080	0.375	0.049	2.852	0.345
**DM**								
Without	Reference				Reference			
With	0.190	0.027	1.368	0.101	0.379	0.050	2.877	0.350
**Hyperlipidemia**								
Without	Reference				Reference			
With	0.000	-	-	0.301	0.000	-	-	0.957
**COPD**								
Without	Reference				Reference			

With	0.425	0.059	3.046	0.397	0.569	0.071	4.514	0.594
**CKD**								
Without	Reference				Reference			
With	0.000	-	-	0.605	0.000	-	-	0.977
**IHD**								
Without	Reference				Reference			
With	0.565	0.079	4.050	0.571	1.163	0.156	8.650	0.862
**CHD**								
Without	Reference				Reference			
With	0.000	-	-	0.556	0.000	-	-	0.975
**Stroke**								
Without	Reference				Reference			
With	1.384	0.342	5.609	0.630	2.199	0.510	9.468	0.278
**Cancer**								
Without	Reference				Reference			
With	0.208	0.030	1.496	0.120	0.570	0.078	4.185	0.582
**Anxiety**								
Without	Reference				Reference			
With	3.641	2.098	6.316	<0.001	3.053	1.921	4.852	<0.001
**Depression**								
Without	Reference				Reference			
With	7.489	5.005	11.207	<0.001	3.053	1.921	4.852	<0.001
**Obesity**								
Without	Reference				Reference			
With	0.000	-	-	0.651	0.000	-	-	0.977
**Season**								
Spring	Reference				Reference			
Summer	0.770	0.418	1.411	0.417	0.739	0.398	1.373	0.355
Autumn	0.930	0.532	1.626	0.824	0.909	0.511	1.618	0.771

Winter	1.555	0.909	2.658	0.096	1.712	0.995	2.945	0.046
**Location**					**Had collinearity with urbanization level**			
Northern Taiwan	Reference				**Had collinearity with urbanization level**			
Middle Taiwan	1.384	0.863	2.219	0.159	**Had collinearity with urbanization level**			
Southern Taiwan	1.144	0.680	1.924	0.569	**Had collinearity with urbanization level**			
Eastern Taiwan	1.973	0.998	3.886	0.052	**Had collinearity with urbanization level**			
Outlets islands	0.000	-	-	0.950	**Had collinearity with urbanization level**			
**Urbanization level**								
1 (The highest)	0.464	0.249	0.864	0.017	0.550	0.276	1.094	0.095
2	0.961	0.560	1.648	0.910	0.909	0.503	1.644	0.777
3	0.352	0.118	1.047	0.063	0.395	0.132	1.180	0.100
4 (The lowest)	Reference				Reference			
**Level of care**								
Hospital center	1.444	0.804	2.589	0.199	1.600	0.842	3.040	0.138
Regional hospital	1.592	0.906	2.795	0.095	1.363	0.755	2.461	0.280
Local hospital	Reference				Reference			

HR= hazard ratio, CI = confidence interval, Adjusted HR: Adjusted variables listed in the table
